# A Mouse Model of Multi-Drug Resistant *Staphylococcus aureus*-induced Ocular Disease

**DOI:** 10.13188/2334-2838.1000026

**Published:** 2016-11-10

**Authors:** Nicole M. Broekema, Inna V. Larsen, Erika S. Naruzawa, Marcin Filutowicz, Aaron W. Kolb, Leandro B. C. Teixeira, Curtis R. Brandt

**Affiliations:** 1 Amebagone, Inc.; 2 Department of Ophthalmology and Visual Sciences, School of Medicine and Public Health, University of Wisconsin-Madison, Wisconsin, USA; 3 Department of Bacteriology, University of Wisconsin-Madison, Wisconsin, USA; 4 Department of Pathobiological Sciences, School of Veterinary Medicine, University of Wisconsin-Madison, Wisconsin, USA; 5 Department of Medical Microbiology and Immunology, School of Medicine and Public Health, University of Wisconsin-Madison, Wisconsin, USA; 6 McPherson Eye Research Institute - University of Wisconsin-Madison, Wisconsin, USA

**Keywords:** *Staphylococcus aureus*, MRSA, Ocular disease, Bacterial keratitis, Mouse model

## Abstract

*Staphylococcus aureus* infection of the cornea is a significant threat to vision. The percentage of bacterial isolates resistant to antibiotics is increasing as is the percentage of infections caused by methicillin resistant isolates. There is a critical need for additional therapeutic approaches and their development will require the use of animal models to test efficacy. Two mouse models of *S. aureus* keratitis have been described but only quantified stromal keratitis (corneal clouding and perforation). We have extended these models using the methicillin resistant *S. aureus* USA300 LAC strain and show that eyelid inflammation and swelling (blepharitis) and corneal neovascularization can be quantified. This expanded model should prove useful in assessing additional effects of antibacterial therapies and additional pathological mechanisms involved in bacterial ocular infection.

## Introduction

Humans carry *Staphylococcus aureus* (*S. aureus*) in numerous body sites [1–3] and it is the most common cause of hospital and community-acquired infections worldwide [5–9]. Community associated methicillin-resistant *S. aureus* (MRSA) infections are estimated to cost $1.4 to 13.8 billion annually [10]. Infection of the eye with *S. aureus* can also cause bacterial keratitis [11–13]. In the past 20 years, the number of ocular MRSA infections has increased worldwide [14–18]. One *S. aureus* strain, MRSA-USA300 (USA300), is common in community acquired infections [19,20]. Symptoms of bacterial keratitis include pain, redness, inflammation, opacity of the affected cornea, and ulceration [21]. Individuals who have undergone ocular surgery, who use contact lenses, and those who have had ocular viral infection or ocular trauma are more susceptible to bacterial keratitis [22–24].

Typically, bacterial keratitis is treated with topical antibiotics [25]. A key issue in bacterial keratitis treatment is that while bacteria rapidly proliferate prior to disease, by the onset of severe symptoms, the bacteria have stopped growing and may have formed an antibiotic-resistant biofilm. In addition to damage from the immune response, non-growing (stationary) phase *S. aureus* produces a number of toxins that contribute to corneal damage [26]. Prompt bactericidal therapy of asymptomatic infection is imperative, but is dependent on when the patient seeks help and the availability of appointments, so this is not always achievable. Many isolates of *S. aureus* are resistant to antibiotics with some strains being resistant to multiple antibiotics [27–32]. New approaches are needed to treat ocular infections caused by antibiotic-resistant *S. aureus* and other bacteria.

Previously, two models of *S. aureus* keratitis were described. Girgis developed a mouse model using *S. aureus* (strain 8325-4) and Zaidi et al. used the USA300 strain [33,34]. However, *S. aureus* 8325-4 is a laboratory strain that carries multiple mutations that may alter the virulence properties of this strain [35,36]. There is also conflicting data on whether this lab-adapted strain can form biofilms [37–40]. Furthermore, these previous studies only scored corneal damage due to stromal keratitis (clouding and perforation). Other pathological manifestations such as blepharitis and corneal neovascularization were not scored. Because other parameters of ocular pathology could be important endpoints in studies of disease mechanisms and evaluating new therapies, we adapted a mouse ocular disease scoring system that we have utilized for antiviral studies [41–48]. In this study, we assessed blepharitis, corneal neovascularization and stromal keratitis in USA300-infected, Ciprofloxacin-treated and untreated mice. This mouse model will be useful for further development and testing of ocular topical antimicrobials and studies on the mechanisms of pathogenesis.

## Methods

### Bacteria

The *S. aureus* USA300 LAC strain was cultured overnight at 37 °C with shaking at 225 rpm in Tryptic Soy Broth. The culture was then centrifuged at 4000 rpm for 10 min, resuspended in 40 ml of phosphate buffered saline (PBS), and centrifuged again at 4000 rpm for 5 min. The pellet was then resuspended in 1 ml of PBS. Colony Forming Units (CFU) of the suspension were determined on SM/2 agar plates (supplemented with 0.5% D-glucose) [49,50]. The inoculum contained 3 × 10^12^ CFU/ml of bacteria.

### Animals

Female A/J mice (4–6 weeks of age) were obtained from Jackson Labs (Bar Harbor, ME) and acclimated to their surroundings for one week prior to infection. For all inoculations, examinations, treatments and sample collections, mice were anesthetized with isoflurane (#57319-47406, Phoenix Pharmaceutical, St. Joseph, MO). The right eyes were examined microscopically prior to infection for corneal defects and those with defects were removed from the study. The remaining mice were then randomly assigned to groups (10 mice each). Under anesthesia, six to ten scratches forming a cross-hatch pattern were made on the cornea using a 30-gauge needle taking care not to puncture the cornea. A 2.5 µL inocula of *S. aureus* USA300 (7.5 × 10^9^ CFU) was applied to the scarified cornea, and the eyelids were manually closed twice over the cornea.

To provide analgesia, the mice were injected subcutaneously with 0.5 mg/kg of extended release Buprenorphine (kindly provided by Dr. Lisa Krugner-Higby, UW-Madison) just prior to corneal scarification. These studies adhered to the ARVO Statement for the Use of Animals in Ophthalmic and Vision Research and NIH guidelines for the use of animals in research and were approved by the University of Wisconsin-Madison IACUC.

### Treatment

A 5 µL drop of 0.3% Ciprofloxacin (NDC 16571-120-50, Pack Pharmaceuticals, Buffalo Grove, IL) or 1% methylcellulose in PBS (vehicle) was applied to the cornea of the infected eye, starting at 4 hours post-infection at 2 hour intervals for a total of 5 treatments per day for 4 days.

### Collection of eye washes and determining number of USA300 viable cells in the washes

On days 1, 2 and 3 post-infection, tear film samples were collected and the number of viable cells of *S. aureus* USA 300 was determined. The infected corneas were flushed with 10 µL of PBS and the wash was then added to 40 µL PBS and kept on ice until samples were serially diluted and spread on SM/2 agar plates. The plates were incubated at 37 °C and colonies counted after a 24 hr incubation period.

### Disease scoring

On days 1 and 3 post-infection, ocular disease severity was scored as previously described, based on three disease parameters-blepharitis, neovascularization, and stromal keratits [42,44,45]. Briefly, blepharitis, or swelling of the eyelid, was scored: 1+, puffy eyelids; 2+, puffy eyelids with some crusting; 3+, eye swollen shut with severe crusting; and 4+, eye completely swollen shut and crusted over. Neovascularization, the growth of blood vessels into the cornea, was scored: 1+, <25% of the cornea involved; 2+, 25% to 50% corneal involvement; and 3+, >50% corneal involvement. Stromal keratitis was scored: 1+, cloudiness, some iris detail visible; 2+, iris detail obscured; 3+, cornea totally opaque; and 4+, corneal perforation.

### Histology

All animals were euthanized at 3 days post-infection. The enucleated eyes were fixed in 4% paraformaldehyde, embedded in paraffin, sectioned, stained with hematoxylin and eosin (H&E), and examined by light microscopy.

### Statistical analysis

Statistical analyses were conducted using Sigma Plot 11.0 (Systat Software, Chicago, IL). At the designated time points, raw scores for each disease parameter were recorded for each mouse in a group. The mean disease scores were calculated for each group from the raw scores and analyzed for statistical significance. Mean peak disease scores (MPDS) were calculated as previously described [42]. The t-test or the Mann-Whitney Rank Sum test was used for pairwise comparisons of the average disease scores and MPDS of groups. P-values < 0.05 were deemed significant unless otherwise stated.

## Results

**Figure 1:**
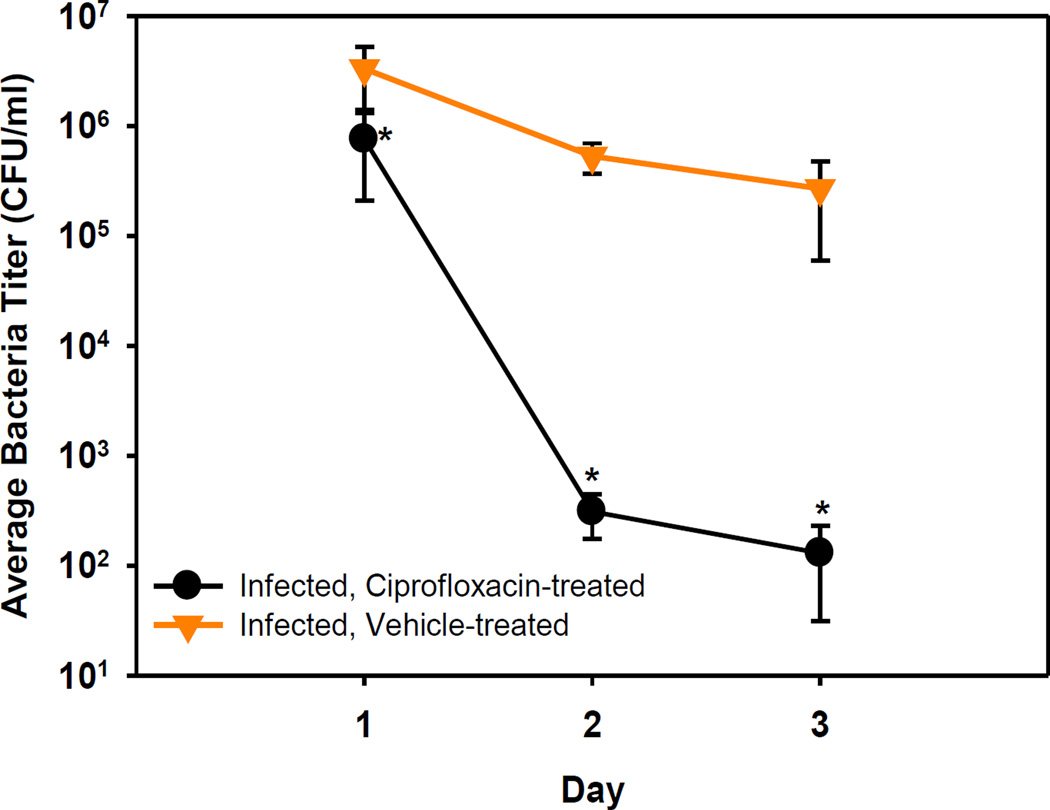
Bacterial titers in eye washes (CFU/ml) on days 1, 2, and 3 post-infection. All data points are the mean ± SEM per group. *p < 0.05.

Bacterial cell numbers from corneal washes varied from 9 × 10^5^ to 5 × 10^6^ CFU/ml at 24 hrs post-infection (Figure 1). At 2 and 3 days post-infection, bacterial cell numbers in the untreated eyes remained in the range of 1 × 10^6^ CFU/ml, whereas bacterial cell numbers in the Ciprofloxacin-treated animals were reduced by 3–4 log__10__. The differences in bacterial titer were significant on all days post-infection (Rank Sum Test, p < 0.05).

**Figure 2:**
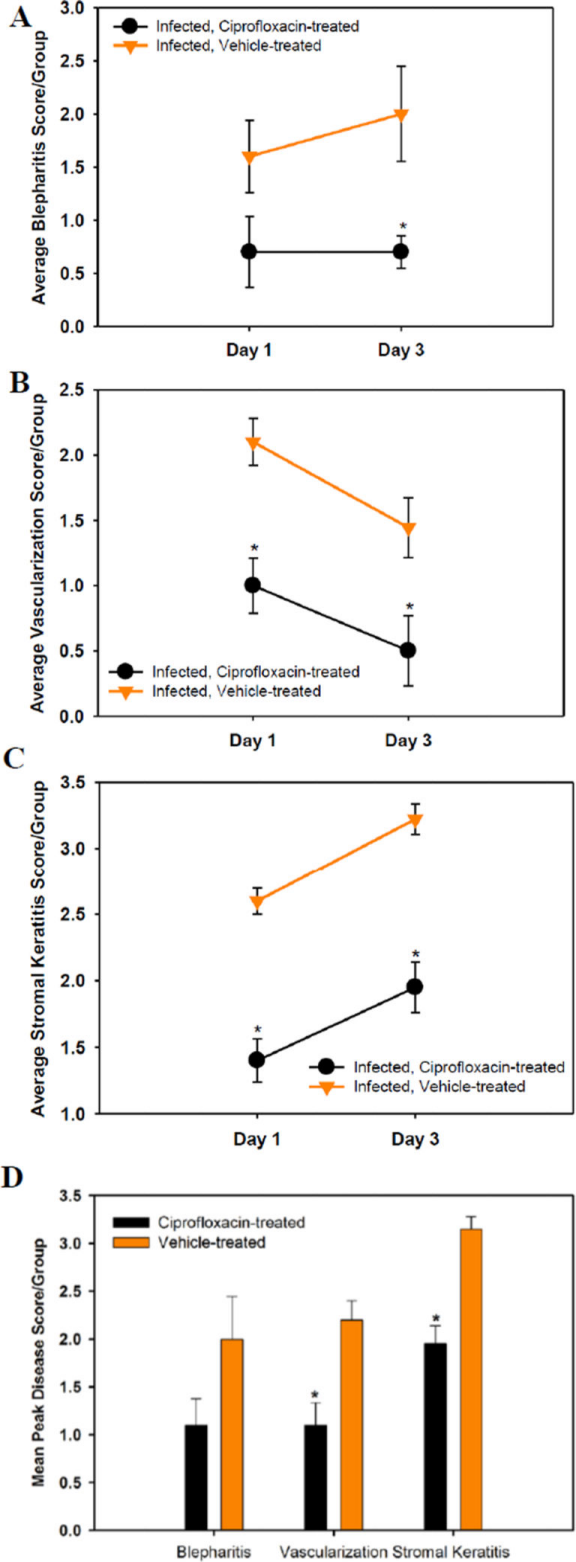
Ocular disease scores of Ciprofloxacin- and vehicle-treated USA300-infected mice and on 1 and 3 days post-infection. A–C represents blepharitis, vascularization, and stromal keratitis respectively. All data points represent the mean ± SEM per group. D Mean peak disease scores (MPDS) for blepharitis, vascularization and stromal keratitis. Scores are the means of the highest scores for each mouse in a group ± SEM. *p < 0.05.

The scores for the severity of blepharitis, corneal neovascularization and stromal keratitis in vehicle and Ciprofloxacin-treated mice are shown in Figure 2. In untreated mice, the blepharitis score was approximately 1.5 on day 1 post-infection and increased to 2.0 on day 3 post-infection (Figure 2A). Blepharitis scores for Ciprofloxacin-treated mice were approximately 0.75 on both days 1 and 3 post-infection (Figure 2A) and were significantly lower for the Ciprofloxacin-treated animals on day 3 post-infection, p < 0.05 (Figure 2A). Corneal neovascularization scores in the vehicle-treated mice were approximately 2.2 on day 1 post-infection and decreased to approximately 1.5 on day 3 post-infection (Figure 2B). In Ciprofloxacin treated mice, neovascularization scores were 1.0 on day 1 post-infection and declined to 0.5 on day 3 post-infection (Figure 2B). Stromal keratitis scores in vehicle-treated mice were approximately 2.6 on day 1 post-infection and increased to 3.3 on day 3 post-infection (Figure 2C). In Ciprofloxacin-treated mice, stromal keratitis scores were approximately 1.4 on day 1 post-infection and increased to 2.0 on day 3 post-infection (Figure 2C). Stromal keratitis and corneal vascularization scores were significantly lower for Ciprofloxacin-treated animals on days 1 and 3 post-infection, p < 0.05 (Figures 2B and 2C). Mean peak disease scores (MPDS) are shown in Figure 2D. For blepharitis, the MPDS were not significantly different but they were lower in the Ciprofloxacin-treated mice. For corneal vascularization and stromal keratitis, the MPDS were significantly lower for the Ciprofloxacin-treated animals, p < 0.05.

### Histopathology

**Figure 3:**
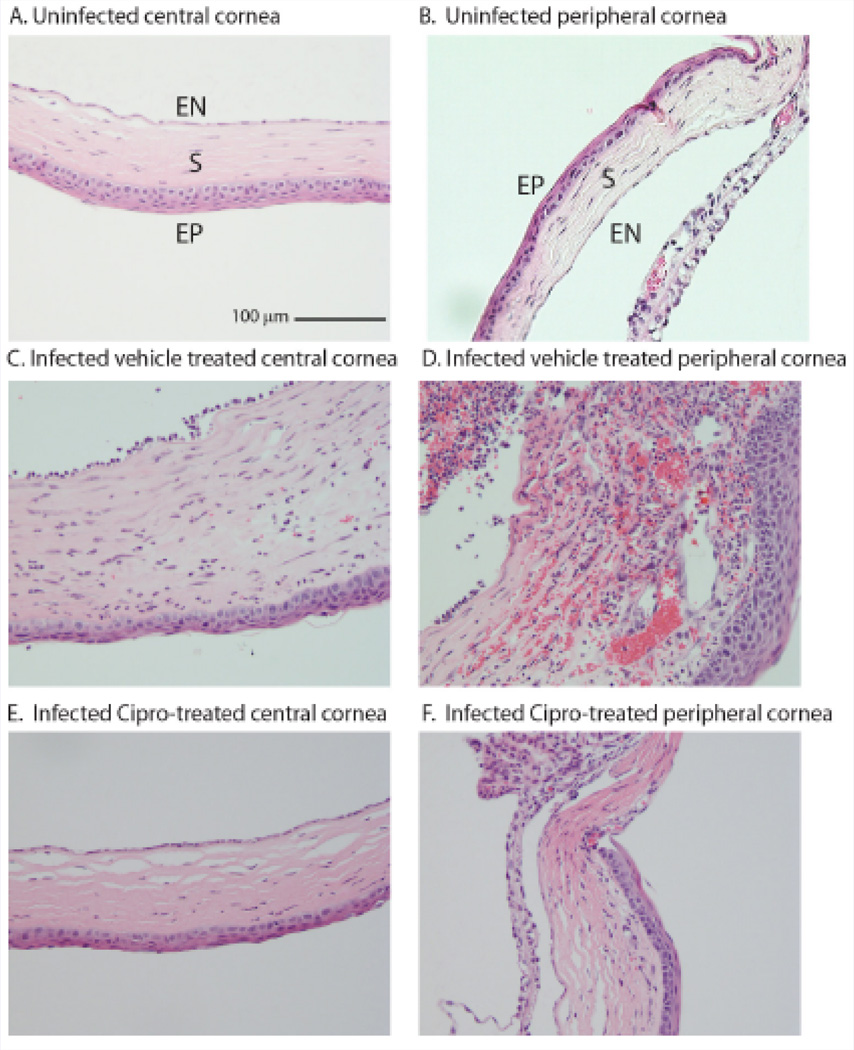
D Infected vehicle treated peripheral cornea. A&B Uninfected, untreated cornea; C&D Infected, vehicle-treated cornea; E&F Infected, 0.3% Ciprofloxacin-treated cornea. H&E, Scale bar 100 µm. EN- corneal endothelium, S - corneal stroma; EP - corneal epithelium.

Eyes infected with *S. aureus* USA300 and treated with vehicle displayed marked corneal epithelial intracellular edema associated with extensive vascularization of the superficial and mid corneal stroma with moderate neutrophilic infiltration, hemorrhage and edema (Figures 3C and 3D). There was also marked hyphema and neutrophilic infiltration in the anterior chamber, especially lining the corneal endothelium and marked infiltration of neutrophils in the iris stroma associated with stromal hemorrhage and formation of a pre-iridal fibrovascular membrane. Eyes infected with *S. aureus* USA300 and treated with ciprofloxacin had mild corneal epithelial keratinization, scant neutrophils dispersed through the superficial stroma, rare neutrophils infiltrating the corneal endothelium and minimal corneal stromal edema (Figures 3E and 3F). All uninfected eyes present a normal microscopic appearance (Figures 3A and 3B).

## Discussion

*S. aureus* keratitis is a significant cause of blindness and the increasing percentage of drug resistant bacteria causing these infections is a major concern. Thus, there is a need for additional antibacterial agents to treat keratitis. Animal models with validated outcome measures are critical for evaluating efficacy at several stages in the drug development process. Mouse models are advantageous in early stage development because they require smaller amounts of test articles than other species commonly used, such as rabbits. Two mouse models of *S. aureus* keratitis were described previously [33,34], but one of these studies used an *S. aureus* strain 8325-4 which is a laboratory strain that has lost the natural ability to form biofilms. Since bacterial keratitis can involve the conjunctiva and eyelids, and corneal neovascularization, these outcomes should be included in any scoring system. We therefore expanded on the previous models and used *S. aureus* USA300 LAC strain that forms biofilms, and have included disease scores for corneal neovascularization and blepharitis. This model should be useful for evaluating the effect of novel antibacterials on eyelid inflammation and swelling and neovascularization of the cornea.

Several studies have reported that MRSA strains are resistant to fluoroquinones, including ciprofloxacin [17,30,32,51]. For example, Freidlin et al. reported that only 14.8% of *S. aureus* isolates were susceptible to ciprofloxacin [17]. We chose to use ciprofloxacin as the positive treatment control in our study because our *S. aureus* USA300 strain is susceptible to the drug. However, other antibiotics could be used as controls depending on the resistance profile of the bacterial isolate being used in the model.

In summary, we have expanded on previous mouse models of *S. aureus* keratitis and included scoring of eyelid swelling and inflammation (blepharitis) and corneal neovascularization. The model should be useful for assessing additional activities of potential new antibacterial drugs, combination therapies to reduce the pathologic inflammatory response, and in studying additional pathologic mechanisms in *S. aureus* keratitis.

## Abbreviations

MRSA: Methicillin Resistant *Staphylococcus aureus*
*S. aureus*: *Staphylococcus aureus*
